# Volatile versus total intravenous anesthesia for 30-day mortality following non-cardiac surgery in patients with preoperative myocardial injury

**DOI:** 10.1371/journal.pone.0238661

**Published:** 2020-09-11

**Authors:** Jungchan Park, Seung-Hwa Lee, Jong-Hwan Lee, Jeong Jin Min, Ji-Hye Kwon, Ah-ran Oh, Keumhee Carriere, Joonghyun Ahn

**Affiliations:** 1 Department of Anesthesiology and Pain Medicine, Samsung Medical Center, Sungkyunkwan University School of Medicine, Seoul, Korea; 2 Department of Cardiology, Samsung Medical Center, Sungkyunkwan University School of Medicine, Seoul, Korea; 3 Statistics and Data Center, Samsung Medical Center, Sungkyunkwan University School of Medicine, Seoul, Korea; 4 Department of Mathematical and Statistical Sciences, University of Alberta, Edmonton, AB, Canada; Cleveland Clinic, UNITED STATES

## Abstract

We evaluated whether volatile anesthetics can improve the postoperative outcomes of non-cardiac surgery in patients with preoperative myocardial injury defined by the cardiac troponin elevation. From January 2010 to June 2018, 1254 adult patients with preoperative myocardial injury underwent non-cardiac surgery under general anesthesia and were enrolled in this study. Patients were stratified into following two groups according to anesthetic agents; 115 (9.2%) patients whose anesthesia was induced and maintained with continuous infusion of propofol and remifentanil (TIVA group) and 1139 (90.8%) patients whose anesthesia was maintainted with volatile anesthetics (VOLATILE group). The primary outcome was 30-day mortality. To diminish the remifentanil effect, a further analysis was conducted after excluding the patients who received only volatile anesthetics without remifentanil infusion. In a propensity-score matched analysis, 30-day mortality was higher in the TIVA group than the VOLATILE group (17.0% *vs*. 9.1%; hazard ratio [HR] 2.60; 95% confidence interval [CI], 1.14–5.93; *p* = 0.02). In addition, the TIVA group showed higher 30-day mortality than the VOLATILE group, even after eliminating the effect of remifentanil infusion (15.8% *vs*. 8.3%; HR 4.62; 95% CI, 1.82–11.74; *p* = 0.001). In our study, the use of volatile anesthetics showed the significant survival improvement after non-cardiac surgery in patients with preoperative myocardial injury, which appears to be irrelevant to the remifentanil use. Further studies are needed to confirm this beneficial effect of volatile anesthetics.

**Clinical trial number and registry URL:** KCT0004349 (www.cris.nih.go.kr)

## Introduction

Cardioprotective effect of volatile anesthetics has been proven in numerous studies [1–4]. So, the use of volatile anesthetics has been recommended to reduce postoperative mortality in patients undergoing major non-cardiac and cardiac surgeries [5–7]. Although a recent MYRIAD trial failed to show the clinical benefits of anesthesia with volatile agents in coronary artery bypass graft surgery [8], the results might be different in non-cardiac surgery since there is no factor which can directly affect the heart such as cardiac manipulation and coronary revascularization.

A leading cause of postoperative mortality in non-cardiac surgery is myocardial injury [9], which was defined as the evidence of elevated cardiac troponin values with at least one value above the 99^th^ percentile upper reference limit [9,10]. As well as postoperative myocardial injury, preoperative cardiac troponin elevation has also been reported to be strongly associated with postoperative mortality in patients undergoing non-cardiac surgery [11–13]. Since volatile anesthetics can reduce not only myocardial infarct size but also cardiac biomarkers [1], the use of volatile anesthetics might be effective to improve clinical outcomes after non-cardiac surgery, especially in patients with preoperative elevation of cardiac troponin.

However, to our knowledge, there has been no study which showed the effect of volatile anesthetics on the postoperative outcomes in non-cardiac surgical patients with preoperative cardiac troponin elevation. Therefore, the aim of our study was to evaluate whether volatile anesthetics can improve 30-day mortality following non-cardiac surgery in patients with preoperative myocardial injury defined by the cardiac troponin elevation.

## Materials and methods

The present study included adult patients who had undergone non-cardiac surgery at Samsung Medical Center (Seoul, Korea) and conducted in accordance with the principles of the Declaration of Helsinki. The study protocol was approved by the Institutional Review Board of Samsung Medical Center (SMC 2019-06-034) and registered by Clinical Research Information Service (KCT0004349). Since our retrospective analysis used only the routinely gathered patient data and had minimal risk of the enrolled patients, the need for individual consent was waived by Institutional Review Board.

### Study population

**Fig 1:**
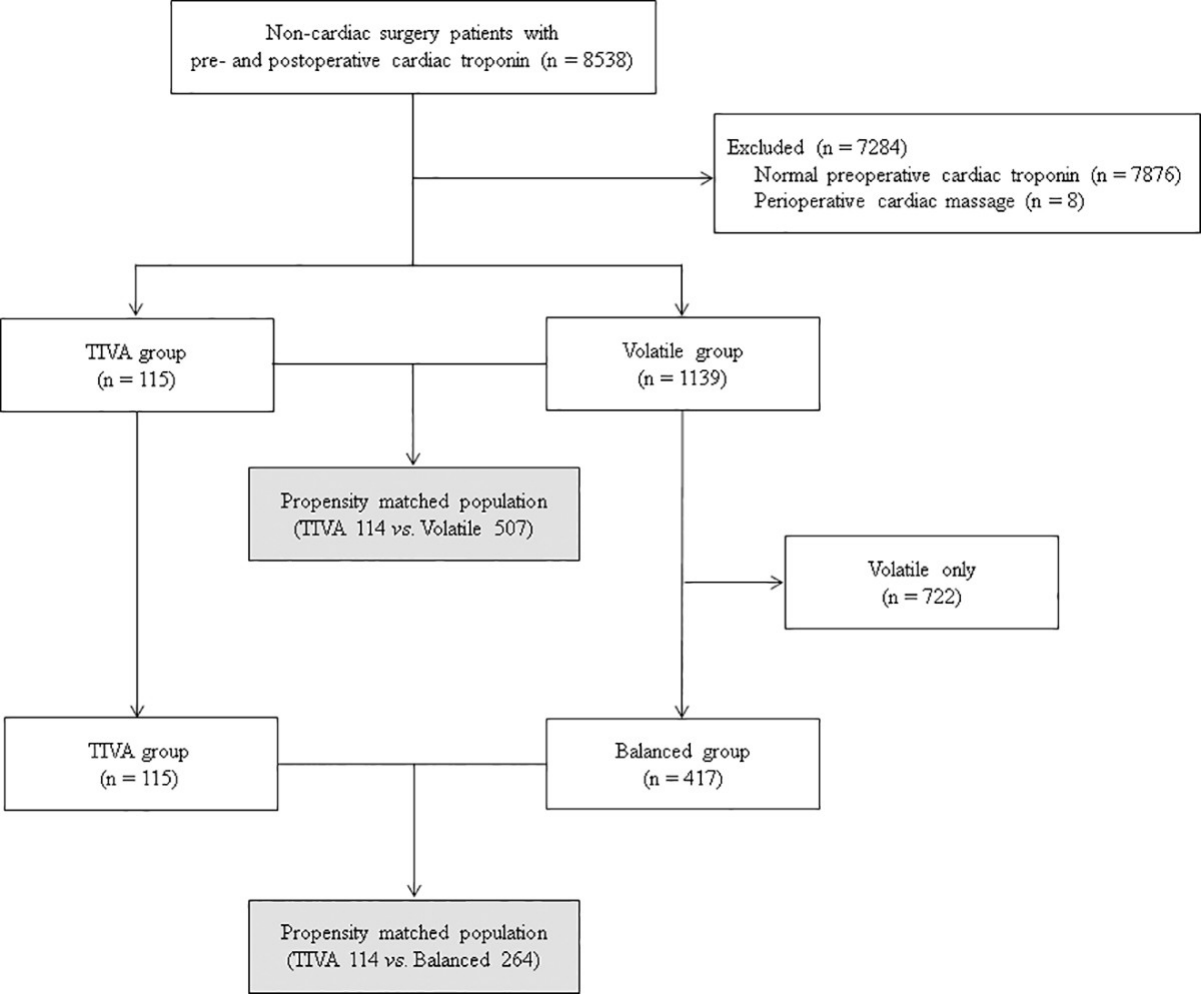
The flowchart of the patients.

From January 2010 to June 2018, all adult patients who underwent non-cardiac surgery under general anesthesia with cardiac troponin measurement before the surgery and repeated measurement within postoperative 7 days at Samsung Medical Center (Seoul, Korea) were initially identified. After excluding the patients with normal preoperative cardiac troponin level or perioperative cardiopulmonary resuscitation, a total of 1254 patients were enrolled in the analysis. According to the anesthetics used during anesthetic maintenance, the enrolled patients were grouped as follows: the TIVA group (*n* = 115), defined as the patients whose anesthesia was induced and maintained with continuous infusion of propofol and remifentanil without using a volatile anesthetic agent and the VOLATILE group (*n* = 1139), defined as the patients whose anesthesia was maintained with volatile anesthetics regardless of induction agents. The choice of anesthetic agents was decided at the attending anesthesiologist’s discretion. The VOLATILE group was further divided according to continuous infusion of remifetanil; 417 patients with remifentanil infusion were grouped into BALANCED group, and 722 patients without remifentanil infusion into ONLY-VOLATILE group (Fig 1). To eliminate the effect remifentanil, the TIVA group was separately compared to the BALANCED group after excluding the ONLY-VOLATILE group. And those three groups were also compared pairwisely.

### Data curation, perioperative management, and cardiac troponin level

Samsung Medical Center operates as a paperless hospital with an electronic medical record system that archives all patients’ information including medical record, prescription, and laboratory findings. The patient selection and data curation of this study were entirely conducted using “Clinical Data Warehouse Darwin-C”, which is another institutional electronic system, designed to search and retrieve de-identified medical records from institutional electronic medical record. In addition to informations from the institutional medical record, mortalities in this system are consistently updated from the national database. After finalizing the list of the patients, independent researchers (J.J. Min and J.-H. Kwon) who were blinded to the anesthetic agents and mortality of the patient organized the extracted data containing baseline characteristics and intraoperative variables into a standardized form.

Anesthetic and postoperative managements were performed according to the institutional protocols based on current guidelines. Perioperative cardiac troponin was not included as a routine practice but was selectively measured at the clinician’s discretion. High-sensitivity cardiac troponin (hs-cTn) I was used for all the patients of this study, and it was measured by a single type of highly sensitive immunoassay (Advia Centaur XP, Siemens Healthcare Diagnostics, Erlangen, Germany). The lowest limit of detection was 6 μg/L, and the normal limit was < 4 μg/L according to the 99th percentile rule, provided by manufacturer [9].

### Study outcomes and definitions

The primary outcome was 30-day mortality. Secondary outcomes included in-hospital mortality, cardiovascular mortality, postoperative further elevation of cardiac troponin, and postoperative acute kidney injury (AKI). Postoperative further elevation of cardiac troponin was defined as higher level of hs-cTn I at any point within 7 days after surgery compared to the baselin measurement. Postoperative AKI was defined based on the Kidney Disease Improving Global Outcomes (KDIGO) criteria using postoperative creatinine level [14]. Previous medical history and the American Society of Anesthesiologists physical status were classified based on preoperative evaluation records [15], and the limitation of daily activity was based on admission note. Perioperative aneamia was defined as hemoglobin <13 g/dL for men and <12 g/dL for women from preoperative evaluation to 48 hours after the surgery [16]. Intraoperative hypotension was defined as mean arterial pressure below 65 mmHg. The risk of surgery was stratified according to the 2014 European Society of Cardiology/Anesthesiology (ESC/ESA) guidelines [17].

### Statistical analysis

Continuous variables are presented as means ± standard deviations (SD), and as frequencies and percentages for categorical variables; data are stratified by treatment group. We used parametric or non-parametric tests as appropriate to compare differences in baseline characteristics. A simple unadjusted Cox regression analysis was used for all outcomes in univariate models. Then, 30-day mortality, in-hospital mortality, and cardiovascular mortality were analyzed using the Cox proportional-hazards regression model, while all other outcomes were analyzed using logistic regression model. Multivariable adjustment initially included all relevant variables, and then backward elimination was performed to fit the final most parsimonious regression model for each outcome. Kaplan-Meier survival curves were constructed and compared with the log-rank test.

To further reduce selection bias and maximize study power while maintaining a balanced confounding variables between the two therapy groups, we used propensity-score matching method. Balance between the two groups was deemed to be achieved when the absolute standardized mean difference (SMD) was less than 10% and the variance ratio was close to 1.0 for each of the covariates. The variates with SMD over 10% after the propensity-score matching were adjusted using the multivariable Cox or logistic regression models, and hazard ratios (HR) or odds ratios (OR), with 95% confidence intervals (CI) between the two therapy groups were reported. Statistical analyses were performed using SPSS 20.0 (IBM Corp., Chicago, IL) and R 3.6.1 (R Development Core Team, Vienna, Austria; http://www.R-project.org/).

## Results

The flowchart of the patients is shown in Fig 1. A total of 8538 adult patients who underwent general anesthesia for non-cardiac surgery with preoperative hs-cTn I measeurement and repeated measeurement within 7 postoperative days were initially identified. After excluding 7876 patients with normal hs-cTn I and 8 patients with perioperative cardiopulmonary resusitation, a total of 1254 patients were left for analaysis. Total of 1254 patients were initially divided according to the use of volatile anesthetics, and 115 (9.2%) and 1139 (90.8%) were grouped into the TIVA and the VOLATILE group, respectively (Table 1). In the VOLATILE group, 722 patients without continuous infusion of remifentanil were identified as the ONLY-VOLATILE group and the remaining 417 patients constituted the BALANCED group.

**Table 1:** Preoperative variables of TIVA and volatile groups.

	Crude population				Propensity-score-matched population			
	TIVA (*n* = 115)	VOLATILE (*n* = 1139)	*p* value	SMD	TIVA (*n* = 100)	VOLATILE (*n* = 386)	*p* value	SMD
Male sex	68 (59.1)	671 (58.9)	0.999	0.4	60 (60.0)	232 (60.1)	0.999	0.2
Age, years	64.2 (±15.7)	65.8 (±14.5)	0.27	10.4	65.6 (±14.9)	65.5 (±14.8)	0.98	2.8
**Previous disease**								
ASA classification			0.73	15.4			0.80	0.3
I	2 (1.7)	14 (1.2)						
II	40 (34.8)	347 (30.5)						
III	61 (53.0)	663 (58.2)						
IV	12 (10.4)	115 (10.1)						
Hypertension*	47 (40.9)	548 (48.1)	0.17	14.6	42 (42.0)	176 (45.6)	0.60	7.3
Diabetes*	36 (31.6)	379 (33.3)	0.75	4.2	30 (30.0)	115 (29.8)	0.999	0.5
PAOD*	10 (8.7)	111 (9.7)	0.84	3.6	8 (8.0)	31 (8.0)	0.999	0.1
Carotid arterial disease*	9 (7.8)	85 (7.5)	0.999	1.4	8 (8.0)	25 (6.5)	0.75	5.9
Stroke*	35 (30.4)	203 (17.8)	0.002	29.8	31 (31.0)	71 (18.4)	0.01	29.5
Cancer*	29 (25.2)	291 (25.5)	0.999	0.8	23 (23.0)	100 (25.9)	0.64	6.8
Chronic kidney disease*	17 (14.8)	269 (23.6)	0.04	22.6	16 (16.0)	102 (26.4)	0.04	25.7
COPD*	22 (19.1)	192 (16.9)	0.63	5.9	21 (21.0)	63 (16.3)	0.34	12.0
Aortic disease*	4 (3.5)	58 (5.1)	0.59	8	2 (2.0)	12 (3.1)	0.50	7.0
PTE/DVT*	5 (4.3)	28 (2.5)	0.37	10.4	5 (5.0)	11 (2.8)	0.45	11.1
**Cardiac disease**								
Coronary artery disease	29 (25.2)	322 (28.3)	0.56	6.9	26 (26.0)	105 (27.2)	0.91	2.7
Heart failure	11 (9.6)	112 (9.8)	0.999	0.9	11 (11.0)	39 (10.1)	0.94	2.9
Arrhythmia	16 (13.9)	190 (16.7)	0.53	7.7	16 (16.0)	49 (12.7)	0.48	9.4
Valve disease	8 (7.0)	56 (4.9)	0.47	8.6	7 (7.0)	23 (6.0)	0.88	4.2
**Preoperative state**								
Limited activity	36 (31.3)	414 (36.3)	0.33	10.7	34 (34.0)	148 (38.3)	0.49	9.0
Ejection fraction <40%	10 (8.7)	84 (7.4)	0.74	4.9	9 (9.0)	32 (8.3)	0.98	2.5
Preop. CRP elevation	60 (52.2)	634 (55.7)	0.54	7	53 (53.0)	205 (5.31)	0.999	0.2
**Previous medication**								
ACEi/ARB	35 (30.4)	292 (25.6)	0.32	10.7	32 (32.0)	106 (27.5)	0.44	9.9
BB	25 (21.7)	260 (22.8)	0.88	2.6	25 (25.0)	94 (24.4)	0.997	1.5
CCB	28 (24.3)	248 (21.8)	0.61	6.1	25 (25.0)	88 (22.8)	0.74	5.2
Antiplatelet	29 (25.2)	358 (31.4)	0.2	13.8	28 (28.0)	110 (28.5)	0.999	1.1
Statin	20 (17.4)	246 (21.6)	0.35	10.6	19 (19.0)	78 (20.2)	0.90	3.0
**Operative risk**			0.05	30.8			0.03	31.0
Low	21 (18.3)	172 (15.1)			15 (15.0)	76 (19.7)		
Intermediate	84 (73.0)	750 (65.8)			76 (76.0)	242 (62.7)		
High	10 (8.7)	217 (19.1)			9 (9.0)	68 (17.6)		
Emergent operation	33 (28.7)	593 (52.1)	< 0.001	49	32 (32.0)	171 (44.3)	0.04	25.5
Perioperative anemia	111 (96.5)	1106 (97.1)	0.95	3.3	97 (97.0)	374 (96.9)	0.999	0.6
**Intraoperative variables**								
Operative duration, hours	2.73 (±2.60)	2.67 (±2.18)	0.79	2.4	2.66 (±2.52)	2.61 (±2.22)	0.83	2.4
Fluid balance	1263.1 (±1464.7)	1451.9 (±2376.4)	0.40	9.6	1226.9 (±1349.9)	1333.3 (±2055.4)	0.62	6.1
Inotropic requirement	36 (31.3)	394 (34.6)	0.55	7	30 (30.0)	118 (30.6)	0.999	1.2
Estimated blood loss, ml	533.0 (±1076.3)	440.0 (±705.9)	0.21	10.1	477.1 (±862.3)	432.4 (±667.6)	0.58	5.8
Intraoperative hypotension	84 (73.0)	851 (74.7)	0.78	3.8	72 (72.0)	290 (75.1)	0.61	7.1
Colloid use	41 (35.7)	587 (51.5)	0.002	32.5	40 (40.0)	161 (41.7)	0.85	3.5
RBC transfusion, packs	0.3 (±1.9)	0.2 (±1.0)	0.9	0.9	0.1 (±0.4)	0.3 (±1.2)	0.16	19.2

Values are n (%) or mean±SD.

Abbreviation: TIVA, total intravenous anesthesia; ASA, American Society of Anesthesiologists; PAOD, peripheral artery occlusion disease; COPD, chronic obstructive pulmonary disease; PTE, pulmonary thromboembolism; DVT, deep vein thrombosis; CRP, C-reactive protein; ACEi, angiotensin-converting enzyme inhibitor; ARB, angiotensin 2 receptor blocker; BB, beta blocker; CCB, calcium channel blocker; RBC, red blood cell; SMD, standard mean difference.

For continuous variables, Wilcoxon rank sum test, paired t test or Wilcoxon signed rank test was used. For categorical variables, x or McNemar test was used

*Variables are not retained for propensity score matching

### TIVA vs. VOLATILE groups

**Fig 2:**
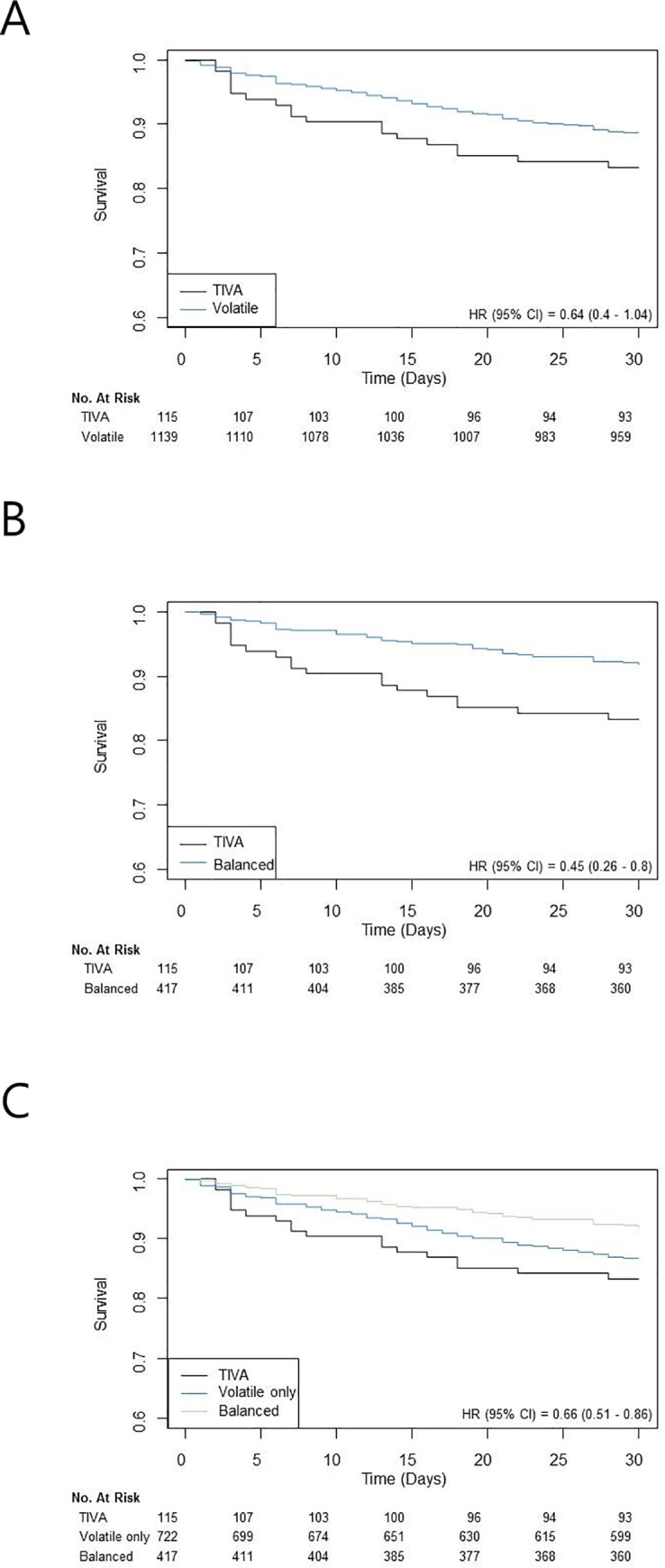
Kaplan-Meier curves for 30-day mortalities of (A) TIVA vs. volatile, (B) TIVA vs. balanced, and (C) TIVA vs. volatile only vs. balanced.

The VOLATILE group showed higher incidence of chronic kidney disease and emergency operation. In the crude population, 30-day mortalities were 16.5% (19/115) in the TIVA group and 11.1% (126/1139) in the VOLATILE group and the TIVA group showed higher 30-day mortality than the VOLATILE group (HR 2.04; 95% CI 1.24–3.35; *p*-value = 0.005) (Table 2). In addition, in-hospital mortality showed similar results between two groups (20.9% vs. 15.3%, HR 1.84; 95% CI 1.19–2.85; *p*-value < 0.001) (Table 2). Surivival curves are presented in Fig 2.

**Table 2:** Clinical outcomes in TIVA versus volatile group comparison.

	TIVA	VOLATILE	Unadjusted HR (95% CI)	*p* value	Adjusted HR (95% CI)	*p* value
**Entire population**	***n* = 115**	***n* = 1139**				
30-day mortality	19 (16.5)	126 (11.1)	1.56 (0.96–3.37)	0.004	2.04 (1.24–3.35)	0.005
In-hospital mortality	24 (20.9)	174 (15.3)	1.66 (1.08–2.54)	0.02	1.84 (1.19–2.85)	<0.001
Postoperative troponin elevation	40 (34.8)	440 (38.6)	0.85 (0.56–1.26)	0.42	1.02 (0.66–1.56)	0.91
AKI, all stage	7 (6.1)	147 (12.9)	0.44 (0.18–0.89)	0.04	0.50 (0.20–1.07)	0.10
*AKI 1*	3 (2.6)	83 (7.3)	0.34 (0.08–0.93)	0.07	0.36 (0.09–1.01)	0.09
*AKI 2*	4 (3.5)	41 (3.6)	0.97 (0.29–2.45)	0.95	1.33 (0.38–3.60)	0.61
*AKI 3*	0	23 (2.0)	-	-	-	-
**Propensity-matched population**	***n* = 100**	***n* = 386**				
30-day mortality	17 (17.0)	35 (9.1)			2.60 (1.14–5.93)	0.02
In-hospital mortality	22 (22.0)	52 (13.5)			1.78 (1.08–2.92)	0.02
Postoperative troponin elevation	37 (37.0)	140 (36.3)			1.06 (0.66–1.68)	0.81
AKI, all stage	7 (7.0)	47 (12.2)			0.44 (0.18–0.95)	0.05
*AKI 1*	3 (3.0)	33 (8.5)			0.31 (0.07–0.90)	0.06
*AKI 2*	4 (4.0)	11 (2.8)			1.25 (0.34–3.64)	0.70
*AKI 3*	0	3 (0.8)			-	-

Values are n (%) or median (IQR)

Abbreviation: TIVA, total intravenous anesthesia; AKI, indicates acute kidney injury; OR, odds ratio.

After propensity-score matching, 100 patients were stratified into the TIVA group and 386 patients into the VOLATILE group (Table 1). Both 30-day and in-hospital mortalities were also higher in the TIVA group in the propensity-score matching analysis (17.0% *vs*. 9.1% HR 2.60; 95% CI 1.14–5.93; *p*-value = 0.02 for 30-day mortality and 22.0% *vs*. 13.5% HR 1.78; 95% CI 1.08–2.92; *p*-value = 0.02 for in-hospital mortality, respectively) (Table 2).

### TIVA vs. BALANCED groups

In comparison of baseline characteristics, the BALANCED group showed higher incidence of hypertension and the previous use of medication such as antiplatelet and statin than the TIVA group (Table 3). The incidence and risk for 30-day and in-hospital mortalities were significantly higher in the TIVA group than in the BALANCED group (16.5% *vs*. 7.9% HR 2.29; 95% CI 1.27–4.12; *p*-value = 0.001 for 30-day mortality and 20.9% *vs*. 9.1% HR 2.54; 95% CI 1.50–4.29; *p*-value = 0.01 for in-hospital mortality, respectively) (Table 4 and Fig 2)

**Table 3:** Preoperative variables of TIVA and balanced groups.

	Crude population				Propensity-score-matched population			
	TIVA (*n* = 115)	BALANCED (*n* = 417)	*p* value	SMD	TIVA (*n* = 97)	BALANCED (*n* = 284)	*p* value	SMD
Male sex	68 (59.1)	249 (59.7)	0.996	1.2	58 (59.8)	161 (56.7)	0.68	6.3
Age, years	64.2 (±15.7)	67.0 (±14.3)	0.08	18.3	65.5 (±15.4)	66.8 (±14.5)	0.44	8.9
**Previous medical history**								
ASA classification			0.47	16.7			0.49	18.2
I	2 (1.7)	6 (1.4)			1 (1.0)	4 (1.4)		
II	40 (34.8)	117 (28.1)			31 (32.0)	73 (25.7)		
III	61 (53.0)	255 (61.2)			54 (55.7)	182 (64.1)		
IV	12 (10.4)	39 (9.4)			11 (11.3)	25 (8.8)		
Hypertension*	47 (40.9)	231 (55.4)	0.01	29.4	40 (41.2)	149 (52.5)	0.07	22.6
Diabetes*	36 (31.6)	145 (34.8)	0.56	7.4	29 (29.9)	93 (32.7)	0.69	6.1
PAOD*	10 (8.7)	48 (11.5)	0.49	9.4	8 (8.2)	28 (9.9)	0.79	5.6
Carotid arterial disease*	9 (7.8)	39 (9.4)	0.75	5.4	8 (8.2)	20 (7.0)	0.87	4.5
Stroke*	35 (30.4)	92 (22.1)	0.08	19.1	33 (34.0)	60 (21.1)	0.02	29.2
Cancer*	29 (25.2)	104 (24.9)	0.999	0.6	24 (24.7)	74 (26.1)	0.90	3.0
Chronic kidney disease*	17 (14.8)	111 (26.6)	0.01	29.5	16 (16.5)	85 (29.9)	0.01	32.2
COPD*	22 (19.1)	74 (17.7)	0.84	3.6	18 (18.6)	43 (15.1)	0.53	9.1
Aortic disease*	4 (3.5)	25 (6.0)	0.41	11.9	3 (3.1)	20 (7.0)	0.25	18.1
PTE/DVT*	5 (4.3)	10 (2.4)	0.42	10.8	3 (3.1)	8 (2.8)	0.999	1.6
**Cardiac disease**								
Coronary artery disease	29 (25.2)	132 (31.7)	0.22	14.3	27 (27.8)	80 (28.2)	0.999	0.7
Heart failure	11 (9.6)	44 (10.6)	0.89	3.3	10 (10.3)	33 (11.6)	0.87	4.2
Arrhythmia	16 (13.9)	70 (16.8)	0.55	8.0	16 (16.5)	53 (18.7)	0.75	5.7
Valve disease	8 (7.0)	23 (5.5)	0.72	6.0	7 (7.2)	18 (6.3)	0.95	3.5
**Preoperative state**								
Limited activity	36 (31.3)	153 (36.7)	0.34	11.4	35 (36.1)	107 (37.7)	0.87	3.3
Ejection fraction <40%	10 (8.7)	33 (7.9)	0.94	2.8	8 (8.2)	18 (6.3)	0.68	7.3
Preop. CRP elevation	60 (52.2)	231 (55.4)	0.61	6.5	51 (52.5)	149 (52.5)	0.999	0.2
**Preoperative medication**								
ACEi/ARB	35 (30.4)	132 (31.7)	0.89	0.03	33 (34.0)	87 (30.6)	0.62	7.2
BB	25 (21.7)	124 (29.7)	0.12	18.4	25 (25.8)	83 (29.2)	0.60	7.7
CCB	28 (24.3)	117 (28.1)	0.5	8.4	26 (26.8)	71 (25.0)	0.83	4.1
Antiplatelet	29 (25.2)	166 (39.8)	0.01	31.5	27 (27.8)	91 (32.0)	0.52	9.2
Statin	20 (17.4)	114 (27.3)	0.04	24	19 (19.6)	64 (22.5)	0.64	7.2
**Operative risk**			0.02	31.9			0.12	25.4
Low	21 (18.3)	64 (15.3)			15 (15.5)	49 (17.3)		
Intermediate	84 (73.0)	271 (65.0)			73 (75.3)	186 (65.5)		
High	10 (8.7)	82 (19.7)			9 (9.3)	49 (17.3)		
Emergent operation	33 (28.7)	160 (38.4)	0.07	20.6	32 (33.0)	100 (35.2)	0.79	4.7
Perioperative anemia	111 (96.5)	404 (96.9)	0.999	2.0	95 (97.9)	273 (96.1)	0.60	10.7
**Intraoperative variables**								
Operative duration, hours	2.73 (±2.60)	2.51 (±1.84)	0.31	9.7	2.59 (±2.44)	2.63 (±1.99)	0.88	1.7
Fluid balance	1263.1 (±1464.7)	1567.5 (±2348.8)	0.19	15.6	1309.9 (±1553.3)	1452.7 (±1903.7)	0.51	8.2
Inotropic requirement	36 (31.3)	114 (27.3)	0.47	8.7	29 (29.9)	85 (29.9)	0.999	0.1
Estimated blood loss, ml	533.0 (±1076.3)	424.2 (±848.9)	0.26	11.1	476.4 (±874.0)	433.2 (±780.2)	0.65	5.2
Intraoperative hypotension	84 (73.0)	299 (71.7)	0.87	3	69 (71.1)	211 (74.3)	0.63	7.1
Colloid use	41 (35.7)	196 (47.0)	0.04	23.2	40 (41.2)	124 (43.7)	0.77	4.9
RBC transfusion, packs	0.3 (±1.9)	0.1 (±0.6)	0.21	9.5	0.1 (±0.4)	0.1 (±0.6)	0.54	7.7

Values are n (%) or mean±SD.

Abbreviation: TIVA, total intravenous anesthesia; ASA, American Society of Anesthesiologists; PAOD, peripheral artery occlusion disease; COPD, chronic obstructive pulmonary disease; PTE, pulmonary thromboembolism; DVT, deep vein thrombosis; CRP, C-reactive protein; ACEi, angiotensin-converting enzyme inhibitor; ARB, angiotensin 2 receptor blocker; BB, beta blocker; CCB, calcium channel blocker; RBC, red blood cell; SMD, standard mean difference.

For continuous variables, Wilcoxon rank sum test, paired t test or Wilcoxon signed rank test was used. For categorical variables, x or McNemar test was used

*Variables retained for propensity score matching

**Table 4:** Clinical outcomes in TIVA versus balanced group comparison.

	TIVA	BALANCED	Unadjusted HR (95% CI)	*p* value	Adjusted HR (95% CI)	*p* value
**Entire population**	***n* = 115**	***n* = 417**				
30-day mortality	19 (16.5)	33 (7.9)	0.79 (0.29–2.75)	0.001	2.29 (1.27–4.12)	0.001
In-hospital mortality	24 (20.9)	38 (9.1)	2.40 (1.43–4.00)	0.001	2.54 (1.50–4.29)	0.01
Postoperative troponin elevation	40 (34.8)	141 (33.8)	1.04 (0.67–1.60)	0.85	1.22 (0.76–1.95)	0.40
AKI, all stage	7 (6.1)	32 (7.7)	0.78 (0.31–1.72)	0.56	0.80 (0.31–1.83)	0.62
*AKI 1*	3 (2.6)	23 (5.5)	0.46 (0.11–1.35)	0.21	0.43 (0.10–1.33)	0.19
*AKI 2*	4 (3.5)	6 (1.4)	2.47 (0.62–8.79)	0.17	3.15 (0.72–12.73)	0.11
*AKI 3*	0	3 (0.7)	-	-	-	-
**Propensity-matched population**	***n* = 97**	***n* = 284**				
30-day mortality	18 (15.8)	22 (8.3)			4.62 (1.82–11.74)	0.001
In-hospital mortality	23 (20.2)	25 (9.5)			2.67 (1.49–4.78)	0.001
Postoperative troponin elevation	40 (35.1)	74 (28.0)			1.26 (0.77–2.05)	0.36
AKI, all stage	7 (6.1)	21 (8.0)			0.68 (0.24–1.61)	0.41
*AKI 1*	3 (2.6)	17 (6.4)			0.31 (0.05–1.12)	0.13
*AKI 2*	4 (3.5)	3 (1.1)			2.99 (0.69–1.31)	0.13
*AKI 3*	0	1 (0.4)			-	-

Values are n (%) or median (IQR)

Abbreviation: TIVA, total intravenous anesthesia; AKI, indicates acute kidney injury; OR, odds ratio.

A separate propensity-score matching was conducted for this population, and all confounding variables were well balanced in this matched set of population. Similarly with the crude population, the TIVA group showed higher incidence and risk for 30-day and in-hospital mortalities than the BALANCED group (15.8% *vs*. 8.3% HR 4.62; 95% CI 1.82–11.74; *p*-value = 0.001 for 30-day mortality and 20.2% *vs*. 9.5% HR 2.67; 95% CI 1.49–4.78; *p*-value = 0.001 for in-hospital mortality, respectively) (Table 4).

### TIVA vs. ONLY-VOLATILE vs. BALANCED groups

In addition, the following three groups of the entire population were compared pariwisely; TIVA group (115/1254, 9.2%) *vs*. volatile only group (722/1254, 57.6%) *vs*. balanced group (417/1254, 33.3%). The baseline characteristics and types of surgery are presented in S1 and S2 Tables, available as Electronic Supplementary Material. In a comparison to the ONLY-VOLATILE group, the TIVA group significantly showed higher risks for 30-day and in-hospital mortalities (16.5% *vs*. 12.9% HR 1.93; 95% CI 1.15–3.22; *p*-value = 0.01 for 30-day mortality and 20.9% *vs*. 19.0% HR 1.79; 95% CI 1.14–2.81; *p*-value = 0.01 for in-hospital mortality, respectively) (S3 Table, available as Electronic Supplementary Material). Additionally, in comparisons between the ONLY-VOLATILE and the BALANCED groups, the incidences of postoperative cardiac troponin elevation and AKI were significantly higher in the ONLY-VOLATILE group (41.4% *vs*. 33.8% HR 0.74; 95% CI 0.57–0.96; *p*-value = 0.03 for cardiac troponin elevation and 15.9% *vs*. 7.7% HR 0.60; 95% CI 0.39–0.92; *p*-value = 0.02 for AKI, respectively) (S3 Table and Fig 2).

## Discussion

Our study showed that, in patients with preoperative myocardial injury, the use of volatile anesthetic agents would be associated with the improved early postoperative mortality regardless of remifentanil infusion.

For the recent few decades, anesthetic management has developed in a way to lower cardiac stressor, and it resulted as a dramatic improvement of anesthesia-related outcomes [18,19]. Among the anesthetic agents, volatile agents have been traditionally recommended as a key intervention to improve survival after major or cardiac surgeries for their cardioprotective effects [5–7]. However, in previous studies, propofol has shown organ-protective effect with anti-inflammatory, immune-modulatory, and antioxidant properties [20,21] and a recent MYRIAD trial also concluded that the actual clinical benefit of volatile anesthetics over TIVA does not exist for the coronary artery bypass graft surgery [8]. In addition, even in previous trials from the non-cardiac surgical patients, the previous studies have focused on the particular type of surgery instead of all types of non-cardiac surgery [1,2,22]. To focuse on the patients who would be benefited from using volatile anesthetics, we enrolled the patients undergoing all kinds of non-cardiac surgery.

In the fourth universal definition of myocardial infarction, myocardial injury is defined as a sole elevation of cardiac troponin without ischemic symptom [10], which was mainly based on the robust evidences for the strong association between postoperative elevation of cardiac troponin and mortality in non-cardiac surgical patients [23–25]. Differently from the postoperative myocardial injury, preoperative cardiac troponin elevation have received less attention and, in some studies, was excluded since it was considered as chronic elevation [23,24,26]. However, several recent studies have suggested that preoperative elevation of cardiac troponin might be also related to increased postoperative mortality in non-cardiac surgery [11–13]. In the present analysis, 30-day mortality were 12% in all population. Considering that mortality within 30 days in patients with myocardial injury in patients with myocardial injury after non-cardiac surgery has been reported as around 10% [9], preoperative cardiac troponin elevation appeared to be also associated with an increase in postoperative mortality in non-cardiac surgical patients. Since there has been no study for the appropriate management of patients with preoperative myocardial injury, our result might suggest a way to improve postoperative mortality in patients with preoperative myocardial injury. In addition, we identified a specific patients’ condition in which volatile anesthetic agents consistently showed a beneficial effect of postoperative outcomes.

Since remifentanil also showed the protective effect against ischemic injury [27–29], 30-day mortaliy was compared between the TIVA and BALANCED groups in our study. After removing the confounding effect from the use of remifentanil, volatile anesthetic agents consistently showed the survival benefit compared to the propofol infusion. In addition, in a pairwise comparison among the TIVA, ONLY-VOLATILE, and BALANCED groups, the patients in the ONLY-VOLATILE group also showed a survival benefit over those in the TIVA group. However, to our thought, further study should be needed to confirm those findings.

Interestingly, the BALANCED group showed lower incidences of postoperative cardial troponin elevation and AKI than the ONLY-VOLATILE group in our analysis. Those results might be caused from the above-mentioned protective effect of remifentanil against ischemic injury [27–29] or the sympathetic block of remifentanil from the intraoperative stimulation [30,31]. However, it would be very hard to conclude since the present study was not designed to compare the remifentanil effect.

This study should be appraised considering several limitations. First, this was a single-center, small-sized, and retrospective study. Therefore, our results might have been affected by confounding factors. And the possibility of bias from hidden or unobserved variables exist despite rigorous statistical adjustments and efforts to include all established contributors. And also, intraoperative time-weighted blood pressure could not be calculated in all patients. Second, hs-cTn measurement was not included as a routine practice, but selectively done in high-risk patients. Although we focused on high-risk patients with elevated cardiac troponin, enrolling only the patients with both pre- and postoperative hs-cTn measurements could have caused selection bias. Third, the use of opioid other than remifentanil and different induction agents in the volatile group were not considered. In addition, the patients in TIVA group all received remifentanil, so individual comparison of volatile anesthetics to propofol could not be made. However, considering the most commonly used combinations of anesthetic agents, our data are more likely to reflect real-world practive. Despite these limitations, this study evaluated the effect of volatile anesthetics on all types of non-cardiac surgery in patients with cardiac troponin elevation and has clinical impacts on anesthetic management of high-risk patients.

## Conclusions

In non-cardiac surgical patients with preoperative myocardial injury, the use of volatile anesthetic agents instead of TIVA showed the significant survival improvement regardless of remifentanil use. However, to confirm our findings, further studies should be needed.

## Supporting information

S1 Table Types of surgery. (DOCX)

S2 Table Preoperative variables. (DOCX)

S3 Table Clinical outcomes. (DOCX)
