# Supplementary material for: Volatile versus total intravenous anesthesia for 30-day mortality following non-cardiac surgery in patients with preoperative myocardial injury
Source: PLoS One. 2020 Sep 11;15(9):e0238661. doi: 10.1371/journal.pone.0238661 (PMC7485855; doi:10.1371/journal.pone.0238661)
Supplement: S1 Table — (DOCX) [file pone.0238661.s001.docx]

**S1 Table. Types of surgery.**

|  | **TIVA (n = 115)** | **ONLY-VOLATILE (n = 722)** | **BALANCED (n = 417)** |
| --- | --- | --- | --- |
| Vascular | 6 (5.2) | 66 (9.1) | 87 (20.9) |
| Abdominal | 32 (27.8) | 365 (50.6) | 120 (28.8) |
| Orthopedics | 22 (19.1) | 161 (22.3) | 98 (23.5) |
| Neuro | 2 (17.4) | 52 (7.2) | 52 (12.5) |
| Otolaryngology, Eye | 30 (26.1) | 50 (6.9) | 48 (11.5) |
| Urology, gynecology | 5 (4.3) | 28 (3.9) | 12 (2.9) |

Values are n (%) or mean±SD.
