# Supplementary material for: Volatile versus total intravenous anesthesia for 30-day mortality following non-cardiac surgery in patients with preoperative myocardial injury
Source: PLoS One. 2020 Sep 11;15(9):e0238661. doi: 10.1371/journal.pone.0238661 (PMC7485855; doi:10.1371/journal.pone.0238661)
Supplement: S2 Table — (DOCX) [file pone.0238661.s002.docx]

|  | **TIVA (n = 115)** | **ONLY-VOLATILE (n = 722)** | **BALANCED (n=417)** | ***P*** |
| --- | --- | --- | --- | --- |
| Male sex | 68 (59.1) | 422 (58.4) | 249 (59.7) | 0.92 |
| Age, years | 64.2 (±15.7) | 65.1 (±14.6) | 67.0 (±14.3) | 0.06 |
| **Previous disease** |  |  |  |  |
| ASA classification |  |  |  | 0.40 |
| I | 2 (1.7) | 8 (1.1) | 6 (1.4) |  |
| II | 40 (34.8) | 230 (31.9) | 117 (28.1) |  |
| III | 61 (53.0) | 408 (56.5) | 255 (61.2) |  |
| IV | 12 (10.4) | 76 (10.5) | 39 (9.4) |  |
| Hypertension | 47 (40.9) | 317 (43.9) | 231 (55.4) | < 0.001 |
| Diabetes | 36 (31.6) | 234 (32.4) | 145 (34.8) | 0.65 |
| PAOD | 10 (8.7) | 63 (8.7) | 48 (11.5) | 0.29 |
| Carotid arterial disease | 9 (7.8) | 46 (6.4) | 39 (9.4) | 0.18 |
| Stroke | 35 (30.4) | 111 (15.4) | 92 (22.1) | < 0.001 |
| Cancer | 29 (25.2) | 187 (25.9) | 104 (24.9) | 0.93 |
| Chronic kidney disease | 17 (14.8) | 158 (21.9) | 111 (26.6) | 0.02 |
| COPD | 22 (19.1) | 118 (16.3) | 74 (17.7) | 0.69 |
| Heart failure | 11 (9.6) | 68 (9.4) | 44 (10.6) | 0.82 |
| Arrhythmia | 16 (13.9) | 120 (16.6) | 70 (16.8) | 0.75 |
| Valve disease | 8 (7.0) | 33 (4.6) | 23 (5.5) | 0.5 |
| Aortic disease | 4 (3.5) | 33 (4.6) | 25 (6.0) | 0.42 |
| PTE/DVT | 5 (4.3) | 18 (2.5) | 10 (2.4) | 0.48 |
| **Cardiac disease** |  |  |  |  |
| Coronary artery disease | 29 (25.2) | 190 (26.3) | 132 (31.7) | 0.12 |
| Heart failure | 11 (9.6) | 68 (9.4) | 44 (10.6) | 0.82 |
| Arrhythmia | 16 (13.9) | 120 (16.6) | 70 (16.8) | 0.75 |
| Valve disease | 8 (7.0) | 33 (4.6) | 23 (5.5) | 0.5 |
| **Preoperative state** |  |  |  |  |
| Limited activity | 36 (31.3) | 261 (36.1) | 153 (36.7) | 0.55 |
| Ejection fraction <40% | 10 (8.7) | 51 (7.1) | 33 (7.9) | 0.76 |
| Preop. CRP elevation | 60 (52.2) | 403 (55.8) | 231 (55.4) | 0.77 |
| **Previous medication** |  |  |  |  |
| ACEi/ARB | 35 (30.4) | 160 (22.2) | 132 (31.7) | < 0.001 |
| BB | 25 (21.7) | 136 (18.8) | 124 (29.7) | < 0.001 |
| CCB | 28 (24.3) | 131 (18.1) | 117 (28.1) | < 0.001 |
| Antiplatelet | 29 (25.2) | 192 (26.6) | 166 (39.8) | < 0.001 |
| Statin | 20 (17.4) | 132 (18.3) | 114 (27.3) | < 0.001 |
| **Operative variables** |  |  |  |  |
| Risk |  |  |  | 0.19 |
| Low | 21 (18.3) | 108 (15.0) | 64 (15.3) |  |
| Intermediate | 84 (73.0) | 479 (66.3) | 271 (65.0) |  |
| High | 10 (8.7) | 135 (18.7) | 82 (19.7) |  |
| Emergent operation | 33 (28.7) | 433 (60.0) | 160 (38.4) | < 0.001 |
| Perioperative anemia | 111 (96.5) | 702 (97.2) | 404 (96.9) | 0.89 |
| **Intraoperative variables** |  |  |  |  |
| Operative duration, hours | 2.73 (±2.60) | 2.77 (±2.35) | 2.51 (±1.84) | 0.17 |
| Fluid balance | 1263.1 (±1464.7) | 1385.0 (±2391.2) | 1567.5 (±2348.8) | 0.31 |
| Inotropic requirement | 36 (31.3) | 280 (38.8) | 114 (27.3) | < 0.001 |
| Estimated blood loss, ml | 533.0 (±1076.3) | 449.2 (±608.7) | 424.2 (±848.9) | 0.39 |
| Intraoperative hypotension | 84 (73.0) | 552 (76.5) | 299 (71.7) | 0.19 |
| Colloid use | 41 (35.7) | 391 (54.2) | 196 (47.0) | < 0.001 |
| RBC transfusion, packs | 0.3 (±1.9) | 0.3 (±1.2) | 0.13 (±0.56) | 0.03 |

**S2 Table. Preoperative Variables.**

Values are n (%) or mean±SD.

ASA, American Society of Anesthesiologists; PAOD, peripheral artery occlusion disease; COPD, chronic obstructive pulmonary disease; PTE, pulmonary thromboembolism ; DVT, deep vein thrombosis ; CRP, C-reactive protein; ACEi, angiotensin-converting enzyme inhibitor; ARB, angiotensin 2 receptor blocker; BB, beta blocker ; CCB, calcium channel blocker ; RBC, red blood cell ; SMD, standard mean difference. For continuous variables, Wilcoxon rank sum test, paired t test or Wilcoxon signed rank test was used. For categorical variables, x or McNemar test was used
