# Supplementary material for: Volatile versus total intravenous anesthesia for 30-day mortality following non-cardiac surgery in patients with preoperative myocardial injury
Source: PLoS One. 2020 Sep 11;15(9):e0238661. doi: 10.1371/journal.pone.0238661 (PMC7485855; doi:10.1371/journal.pone.0238661)
Supplement: S3 Table — (DOCX) [file pone.0238661.s003.docx]

**S3 Table. Clinical Outcomes**

|  | n (%) | Unadjusted HR (95% CI) | *P*-value | Adjusted HR (95% CI) | *P*-value |
| --- | --- | --- | --- | --- | --- |
| **30-day mortality** |  |  |  |  |  |
| *TIVA* | 19 (16.5) | 1 |  | 1 |  |
| *ONLY-VOLATILE* | 93 (12.9) | 1.86 (1.13-3.06) | 0.01 | 1.93 (1.15-3.22) | 0.01 |
| *BALANCED* | 33 (7.9) | 2.28 (1.30-4.02) | 0.004 |  |  |
| *VOLATILE vs BALANCED* |  | 0.60 (0.40-0.89) | 0.01 | 0.84 (0.56-1.27) | 0.41 |
| **In-hospital mortality** |  |  |  |  |  |
| *TIVA* | 24 (20.9) | 1 |  | 1 |  |
| *ONLY-VOLATILE* | 137 (19.0) | 1.44 (0.93-2.22) | 0.1 | 1.79 (1.14-2.81) | 0.01 |
| *BALANCED* | 38 (9.1) | 2.41 (1.44-4.02) | < 0.001 |  |  |
| *VOLATILE vs BALANCED* |  | 0.60 (0.42-0.86) | 0.01 | 0.73 (0.50-1.06) | 0.10 |
| **Postoperative troponin elevation** |  |  |  |  |  |
| *TIVA* | 40 (34.8) | 1 |  | 1 |  |
| *ONLY-VOLATILE* | 299 (41.4) | 0.75 (0.50-1.14) | 0.18 | 0.99 (0.64-1.53) | 0.97 |
| *BALANCED* | 141 (33.8) | 1.04 (0.68-1.16) | 0.85 |  |  |
| *VOLATILE vs BALANCED* |  | 0.72 (0.56-0.93) | 0.01 | 0.74 (0.57-0.96) | 0.03 |
| **AKI, all stage** |  |  |  |  |  |
| *TIVA* | 7 (6.1) | 1 |  | 1 |  |
| *ONLY-VOLATILE* | 115 (15.9) | 0.34 (0.16-0.75) | 0.01 | 0.48 (0.19-1.04) | 0.09 |
| *BALANCED* | 32 (7.7) | 0.78 (0.33-1.82) | 0.56 |  |  |
| *VOLATILE vs BALANCED* |  | 0.43 (0.29-0.65) | <0.001 | 0.60 (0.39-0.92) | 0.02 |
| ***AKI 1*** |  |  |  |  |  |
| *TIVA* | 3 (2.6) | 1 |  | 1 |  |
| *ONLY-VOLATILE* | 60 (8.3) | 0.30 (0.09-0.96) | 0.04 | 0.37 (0.09-1.06) | 0.11 |
| *BALANCED* | 23 (5.5) | 0.46 (0.14-1.56) | 0.21 |  |  |
| *VOLATILE vs BALANCED* |  | 0.64 (0.38-1.04) | 0.08 | 0.82 (0.48-1.37) | 0.45 |
| ***AKI 2*** |  |  |  |  |  |
| *TIVA* | 4 (3.5) | 1 |  | 1 |  |
| *ONLY-VOLATILE* | 35 (4.8) | 0.70 (0.25-2.03) | 0.52 | 1.17 (0.33-3.23) | 0.78 |
| *BALANCED* | 6 (1.4) | 2.47 (0.68-8.90) | 0.17 |  |  |
| *VOLATILE vs BALANCED* |  | 0.29 (0.11-0.64) | 0.01 | 0.37 (0.14-0.85) | 0.03 |
| ***AKI 3*** |  |  |  |  |  |
| *TIVA* | 0 | 1 |  | 1 |  |
| *ONLY-VOLATILE* | 20 (2.8) | - | - | - | - |
| *BALANCED* | 3 (0.7) | - | - | - | - |
| *VOLATILE vs BALANCED* |  |  |  | - | - |

Values are n (%) or median (IQR). AKI indicates acute kidney injury
